# Fungemia and Other Fungal Infections Associated with Use of *Saccharomyces boulardii* Probiotic Supplements

**DOI:** 10.3201/eid2708.210018

**Published:** 2021-08

**Authors:** Juha Rannikko, Ville Holmberg, Matti Karppelin, Pertti Arvola, Reetta Huttunen, Eero Mattila, Niina Kerttula, Teija Puhto, Ülle Tamm, Irma Koivula, Risto Vuento, Jaana Syrjänen, Ulla Hohenthal

**Affiliations:** Tampere University, Tampere, Finland (J. Rannikko);; Tampere University Hospital, Tampere (J. Rannikko, M. Karppelin, P. Arvola, R. Huttunen, J. Syrjänen);; University of Helsinki, Helsinki, Finland (V. Holmberg);; Helsinki University Hospital, Helsinki (V. Holmberg, E. Mattila);; Oulu University Hospital, Oulu, Finland (N. Kerttula, T. Puhto);; Kuopio University Hospital; Kuopio Finland (Ü. Tamm, I. Koivula);; Fimlab Laboratories, Tampere (R. Vuento);; Turku University Hospital, Turku, Finland (U. Hohenthal)

**Keywords:** fungemia, fungal infections, fungi, Saccharomyces boulardii, yeast, probiotic supplements, probiotics, live microorganisms, Finland

## Abstract

Because of widespread use of probiotics, their safety must be guaranteed. We assessed use of *Saccharomyces boulardii* probiotic yeast from medical records for patients who had *Saccharomyces* fungemia or other clinical *Saccharomyces* culture findings. We evaluated all *Saccharomyces* sp. findings at 5 university hospitals in Finland during 2009–2018. We found 46 patients who had *Saccharomyces* fungemia; at least 20 (43%) were using *S. boulardii* probiotic. Compared with a control group that had bacteremia or candidemia, the odds ratio for use of an *S. boulardii* probiotic was 14 (95% CI 4–44). Of 1,153 nonblood culture findings, the history for 125 patients was checked; at least 24 (19%) were using the probiotic (odds ratio 10, 95% CI 3–32). This study adds to published fungemia cases linked to use of *S. boulardii* probiotic and sheds light on the scale of nonblood *Saccharomyces* culture findings that are also linked to use of this probiotic.

## Abstract 2

Use of these supplements should be considered carefully for patients whose gastrointestinal tract integrity might be compromised.

Probiotics are live microorganisms intended to provide health benefits when consumed (*1*). Typically, the endpoint in randomized controlled trials of probiotics has been the prevention of diarrhea or faster alleviation of diarrhea symptoms (*2*). Regarding their safety, serious adverse effects have been rare in probiotic studies (*3*). However, the adverse effects have not been fully reported (*4*). In 1 trial in which a multispecies probiotic preparation was given to patients who had severe acute pancreatitis, the mortality rate was higher in the probiotic arm (*5*). Nevertheless, the use of probiotics is common. According to the 2012 National Health Interview Survey in the United States, 1.6% of adults had used prebiotics or probiotics in the preceding 30 days (*6*).

*Saccharomyces cerevisiae* var. *boulardii* is a yeast that is used as a probiotic. In hospitals in the United States, the use of *S. cerevisiae* var. *boulardii* has been common, especially among elderly patients (*7*). This strain is difficult to distinguish microbiologically from *S. cerevisiae* because they have >99% genomic relatedness (*8*). Thus, in everyday clinical laboratory work, the *S. cerevisiae* var. *boulardii* strain is identified as either *Saccharomyces* sp*.* or *S. cerevisiae*. A review from 2005 considered *S. cerevisiae* var. *boulardii* to be the etiologic agent of *Saccharomyces* fungemia if the patient received treatment with a probiotic containing *S. cerevisiae* var. *boulardii* or if a molecular typing method confirmed the identification of this yeast (*9*). The authors found 37 cases in the literature. We found an additional 14 reports, including 22 cases of *Saccharomyces* fungemia with the same diagnostic method published after this review (*10**–**23*). Thus, before our study, 59 cases of fungemia with a link to the use of the probiotic had been published. All of these cases have been either individual cases or small cases series (<7 cases) without any systematic approach to quantify the problem.

Furthermore, besides fungemia, there are few reports on other clinically relevant microbiological findings for this yeast (i.e., in abscesses, ascites fluid, or the pleural cavity). The meta-analysis we mention listed 20 cases of findings other than fungemia (*9*). These findings are useful because they might also lead to a change in antimicrobial treatment.

We conducted a retrospective registry study (case series) at 5 university hospitals in Finland to evaluate the use of the *S. cerevisiae* var. *boulardii* probiotic in patients who had *Saccharomyces* fungemia or another clinical culture finding for this yeast. To evaluate the association between probiotic use and subsequent findings, we compared use of *S. cerevisiae* var. *boulardii* for patients who had a *Saccharomyces* infection with use of *S. cerevisiae* var. *boulardii* for patients who had an infection caused by another etiologic agent, such as bacteria or *Candida* sp.

## Methods

### Background

Finland has 5 university hospitals that are secondary referral centers of their catchment areas and tertiary referral centers for other hospital districts. Their combined catchment areas cover more than half population of Finland (3.29 million of 5.6 million persons). All university hospitals use the same register (SAI-registry; Neotide Ltd, https://www.neotide.fi), in which the local clinical microbial laboratory data are collected. These data cover all blood culture data and most of all other clinical microbial culture data of the catchment area of the university hospital.

### Patient Data

At least 1 infectious diseases specialist in every university hospital collected the clinical data from the medical records for all blood culture-positive cases found in the register that were identified as *Saccharomyces* sp. or *S. cerevisiae*. The use of the *S. cerevisiae* var. *boulardii* probiotic was defined as use at the time of the positive culture or in the preceding 7 days. Data were collected on use during the preceding 3 months. If the medical record was not available, the case-patient was classified as not using a probiotic. The Quick SOFA score and the definition of septic shock were based on the Sepsis‐3 definitions (*24*). The McCabe score was determined as reported by McCabe and Jackson (*25*). Data collected for case-patients who had nonblood cultures were age, sex, malignancy, digestive tract disease, use of probiotics, use of antifungal medication at the time of the culture, and possible change of medication resulting from finding of *Saccharomyces* sp. The most recent 25 cases of nonblood culture findings in each hospital district were evaluated (excluding case-patients who had positive fecal samples). Isolates were obtained from routine laboratory bacterial and fungal cultures. The anatomic site of the culture was collected from the local hospital microbial registry (SAI) for all culture-positive cases. Abdominal sites were those in which culture was taken from, for example, ascites fluid, a biliary drainage catheter, or abscess drainage fluid, but not from skin or wound secretions. Oral and respiratory tract samples were from sinus drainage, bronchial lavage cultures, and pleural drainage. Other sites included samples from perianal abscesses, mediastinum, and urine.

### Control Group

To evaluate the practice of probiotic use in the hospital ward in which the patient who had a *Saccharomyces* finding was given treatment, a control group was obtained from the same SAI register. For every *Saccharomyces* fungemia case-patient, 2 blood culture-positive patients (1 chronologically closest before and 1 after) from the same ward as the case were selected. For every clinical culture sample (other than blood), there was 1 chronologically closest positive culture sample from the same ward as the case-patient who served as a control. Data collected for the controls were date, ward, microbe, age, sex, malignancy, digestive tract disease, and *S. cerevisiae var. boulardii* probiotic use at the time of the positive culture or in the previous 3 months.

### Statistical Analysis

We used SPSS version 22.0 software (IBM Corp., https://www.ibm.com) for statistical analyses. The study was centrally approved by the Ethics Committee of Tampere University Hospital, Tampere, Finland. The requirement for informed consent was waived.

## Results

### Blood Cultures

There were 46 patients with a positive blood culture for *Saccharomyces* in the 5 hospitals during between January 2009‒December 2018. The median age of case-patients was 68 (range 30–93) years and a male predominance (63%). The most common underlying condition was a digestive tract disease (59%). There was a medical record confirming the use of the *S. cerevisiae* var. *boulardii* probiotic on the day of the blood culture or during the preceding 7 days for 20 case-patients (43%). Medical records were not available for 10 case-patients (22%), and these were classified as nonusers.

Of the 20 case-patients, 17 were using *S. cerevisiae* var. *boulardii* probiotic on the day of the blood culture and 3 case-patients had already stopped using it (2 patients on the day before and 1 patient 5 days earlier). Five additional case-patients had used the probiotic in the preceding 3 months, of whom 1 patient had already stopped using it 26 days earlier. For 4 case-patients, the time when the use of the probiotic was terminated could not be determined. Most case-patients (16/20, 80%) received the *S. cerevisiae* var. *boulardii* probiotic in the hospital, 3 case-patients in some other facility, and 1 case-patient was using it at home. All *S. cerevisiae* var. *boulardii* probiotics found in the medical records were from the same strain (Precosa; Biocodex Ltd., https://www.biocodex.com). We provide characteristics, underlying diseases, and severity of the disease for these patients (Tables 1,2).

**Table 1:** Characteristics of 46 case-patients who had *Saccharomyces* fungemias in 5 hospital districts, Finland, January 1, 2009‒December 31, 2018*

Patient no.	Hospital district and case no.	Age, y/sex	Year of fungemia	Medical record of *S. cerevisiae* var. *boulardii* probiotic use in previous 3 mo	Days alive after fungemia	Central venous catheter	GI tract disease	Antimicrobial drug before fungemia	Change in medication because of fungemia	Main reason for hospital stay
1	A1	56/M	2016	Yes	>90	No	Duodenal ulcer	Yes	Yes	Antimicrobial drugs and NSAIDs after tooth surgery led to duodenal ulcer and fungemia
2	A2	42/M	2013	No	>90	No	Achalasia, PEG tube, duodenal ulcer	Yes	Yes	Vomiting, losing weight, duodenal ulcer
3	A3	42/M	2010	No	>90	No	No	No	Yes	Intravenous drug user who had fungemia
4	A4	40/M	2018	No	>90	No	No	No	Yes	Methadone substitution therapy for patient who had fever and was feeling ill
5	A5	77/F	2011	Yes	2	No	PEG tube	Yes	Yes	Spinocellular carcinoma patient who had problems with PEG tube
6	B1	67/M	2009	Yes	>90	Yes	Pancreatitis	Yes	Yes	Necrotizing pancreatitis
7	B2	66/M	2010	Yes	0	No	PEG tube	Yes	No	PEG tube not in place
8	B3	52/M	2012	NA	>90	No	No	No	No	HIV positive with illicit drug use in ED and high fever and diarrhea
9	B4	67/M	2013 (4 different days)	Yes	7	Yes	Chronic pancreatitis with pseudocysts	Yes	Yes	Pseudocyst rupturation into lung
10	B5	88/M	2013	Yes	19	NA	Bowel obstruction and diverticulosis	NA	NA	Diarrhea after antimicrobial drug use that later turned into an occlusion
11	B6	60/M	2014	NA	>90	NA	No	Yes	No	Alcohol abuse, streptococcal septicemia, and bronchopleural fistula
12	B7	70/F	2015	Yes	14	No	Local intestinal thickening of unknown reason	Yes	Yes	Unspecified neurologic disease that lead to cachexia
13	B8	63/M	2015	Yes	26	No	Stomach cancer	Yes	Yes	Lung abscess treated with lobectomia
14	B9	91/F	2015	Yes	39	NA	No	Yes	NA	Varicose ulcer
15	B10	75/F	2016	Yes	>90	No	Pancreatic cancer	Yes	Yes	Recurrent cholangitis
16	B11	67/F	2017	NA (suspected)	>90	NA	PEG tube	NA	NA	Multiple sclerosis, PEG tube, diarrhea
17	B12	55/M	2018	Yes	0	No	No	Yes	No	Alcohol abuse with delirium and *Clostridium difficile* diarrhea
18	B13	86/F	2018	Yes	0	No	Suspected Crohn disease and diverticulosis	Yes	No	*Staphylococcus aureus* infection after femoro-popliteal reconstruction
19	C1	88/F	2016	Yes	0	No	Colitis ulcerosa	Yes	No	Deep venous thrombosis and fasciotomia
20	C2	77/M	2016	No	1	No	No	Yes	No	Alzheimer patient with aspiration pneumonia
21	C3	85/M	2016	NA	82	No	Colon carcinoma and biliary duct obstruction	Yes	Yes	Liver abscess and septicemia due to *Streptococcus* sp., *Lactobacillus*, sp., use of Rhamnonosus, and *Saccharomyces* sp.
22	C4	39/M	2015	Yes	>90	No	Crohn disease	Yes	Yes	Relapse of Crohn disease and spondylitis of cervical spine
23	C5	80/F	2012	Yes	>90	No	Nasogastric tube	Yes	Yes	Fever, abdominal pain, and vomiting after hip surgery
24	C6	91/M	2010	Yes	2	No	No	Yes	No	Severe pneumonia
25	D1	60/M	2017	No	>90	Yes (PICC)	Bowel obstruction	Yes	Yes	Bowel obstruction due to adhesion
26	D2	84/M	2017	Yes	1	No	No	Yes	No	Pneumonia, sepsis, and multiple organ failure
27	D3	76/F	2017	Yes	0	Yes	Cholecystitis and bowel ischemia	Yes	No	Bowel ischemia and sepsis
28	D4	68/M	2017	No	>90	Yes	Esophageal carcinoma	Yes	No	Esophageal carcinoma surgery and diarrhea
29	D5	80/M	2017	NA	NA	NA	NA	NA	NA	Treated in a district hospital
30	D6	78/F	2012	Yes	>90	Yes	Perforation of sigma	Yes	Yes	Sigma operation and wound infection
31	D7	39/M	2014	No	>90	No	No	No	Yes	Alcohol abuse in ED with empyema and sepsis
32	D8	72/F	2015	Yes	NA	No	Pancreatic cancer	Yes	Yes	Pancreatic cancer
33	D9	45/M	2010	No	>90	Yes	Necrotizing pancreatitis	Yes	Yes	Necrotizing pancreatitis with necrosis in multiple other organs
34	D10	67/F	2014	No	>90	No	No	Yes	Yes	Heart and kidney failure
35	D11	83/F	2015	Yes	NA	Yes	Intestinal perforation	Yes	Yes	Intestinal perforation and abscesses
36	D12	58/F	2018	Yes	>90	No	Diverticulitis	Yes	Yes	Diverticulitis and intestinal perforation
37	E1	68/M	2011	Yes	>90	No	No	Yes	Yes	Alcohol abuse and urinary tract infection
38	E2	83/M	2013	NA	14	NA	NA	NA	NA	NA
39	E3	72/F	2015	No	0	Yes	Prepyloric ulcus	Yes	No	Ulcus, peritonitis, and sepsis
40	E4	30/F	2015	NA	10	NA	NA	NA	NA	Treated in a district hospital
41	E5	52/M	2015	Yes	21	No	Biliary tract obstruction	Yes	No	Biliary tract obstruction
42	E6	72/F	2016	Yes	>90	No	Radiation colitis and multiple fistulas	Yes	No	Carcinoma of cervix and fever
43	E7	39/M	2016	No	>90	No	No	No	No	Intravenous drug user with dyspnea
44	E8	59/M	NA	NA	NA	NA	NA	NA	NA	Treated in a district hospital
45	E9	72/M	NA	NA	NA	NA	NA	NA	NA	Treated in a district hospital
46	E10	93/M	NA	NA	NA	NA	NA	NA	NA	Treated in a district hospital

*ED, emergency department; GI, gastrointestinal; NA, medical records not available; NSAIDs, nonsteroidal antiinflammatory drugs; PEG, percutaneous endoscopic gastrostomy; PICC, peripherally inserted central catheter.

**Table 2:** Characteristics of 46 case-patients who had Saccharomyces fungemia in 5 hospital districts, Finland, January 1, 2009‒December 31, 2018*

Characteristic	Value
No. patients	46
Median age, y (range)	68 (30–93)
Sex	29 (63)
M	29 (63)
F	17 (37)
Use of *S. cerevisiae* var. *boulardii* probiotic in preceding 3 mo†	25/46 (54)
Use of *S. cerevisiae* var. *boulardii* probiotic in preceding 7 d†	20/46 (43)
Use of *S. cerevisiae* var. *boulardii* probiotic in preceding 7 d in control group‡	4/76 (5)
Central venous catheter	8 (17)
Use of antimicrobial drugs in preceding 4 weeks	33 (72)
Change in antimicrobial drugs because of fungemia	23 (50)
Underlying diseases	
Digestive tract	27 (59)
Neurologic	11 (24)
Cardiovascular	8 (17)
Solid tumor with metastasis	6 (13)
Diabetes mellitus (any type)	6 (13)
Pulmonary	5 (11)
Liver	4 (9)
Rheumatic	4 (9)
Chronic kidney§	3 (7)
McCabe score†	
No or nonfatal underlying disease	22 (48)
Ultimately fatal underlying diseases (<5 y)	9 (20)
Rapidly fatal underlying diseases (<1 y)	5 (11)
Severity of disease	
qSOFA score >2 at time of fungemia	16 (35)
Septic shock at time of fungemia	6 (13)
Death by day 7 after fungemia	10 (22)

*Values are no. (%) or no. positive/no. tested (%) unless otherwise indicated. †Medical records were not available for 10 case-patients. ‡Medical records were not available for 6 control case-patients. §History of creatinine level >120 μmol/L.

Antimicrobial drugs were commonly used by the patients (72%) during 4 weeks preceding the fungemia. Antifungal treatment was commenced or changed because of *Saccharomyces* fungemia for 23 patients (50%). For an additional 8 patients (17%), the culture result came after the patient had died. Case-fatality rates by day 7 were 22% (10 patients) and by day 28 were 37% (17 patients). Of patients who died by day 28, 6 patients had an ultimately fatal disease (McCabe score 2) and 5 patients had a rapidly fatal disease (McCabe score 3).

### Nonblood Cultures

There were 1,153 nonblood *Saccharomyces* culture findings (fecal samples excluded). There was considerable variation between hospital districts in numbers of the microbial cultures and anatomic sites from which cultures were obtained (Table 3). We evaluated use of probiotics for 125 case-patients. Medical records were not available for 6 of them. Use of *S. cerevisiae* var. *boulardii* probiotic was confirmed for 24 case-patients (19%). This finding was divided by the anatomic site as follows: 17 (21%) of 82 from the abdominal region, 4 (13%) of 30 from the oral or respiratory tract, and 3 (23%) of 13 from other sites. Antifungal medication was already in use at the time of culture for 38% (47/125, the medical record was not available for 1 case-patient) of the case-patients. This finding led to a modification of the antifungal medication in for 35% (44/125, medical records not available for 2 case-patients) of the case-patients.

**Table 3:** Saccharomyces clinical culture findings (excluding fungemias), Finland, January 1, 2009‒December 31, 2018*

Characteristic	All				University hospital district		
	Total	Helsinki	Tampere		Turku	Oulu	Kuopio
Catchment area in 2017	3,310,000	1,650,000	530,000		480,000	400,000	250,000
Inpatient days in 2017	1,814,183	784,252	307,484		294,834	191,612	236,001
Patients who had clinical findings	1,344	649	30		215	285	165
Abdominal region	205	67	21		8	76	33
Oral or respiratory tract	676	387	6		71	137	75
Fecal	191	53	1		130	4	3
Other or unspecified	272	142	2		6	68	54
Patients who had medical record of *Saccharomyces cerevisiae var. boulardii* probiotic per clinical finding†	24/125 (19)	3/25 (12)	6/25 (24)		4/25 (16)	4/25 (16)	7/25 (28)
Abdominal region	15	2	4		1	4	4
Oral or respiratory tract	4	0	1		3	0	0
Other or unspecified	5	1	1		0	0	3
Patients who had medical record of *S. cerevisiae var. boulardii* probiotic in control group	3/123 (2)	0/25 (0)	1/25 (4)		0/23 (0)	0/25 (0)	2/25 (8)
No. patients who had change of antimicrobial drugs because of finding of *Saccharomyces* sp.‡	44/125 (35)	11/25 (44)	3/25 (12)		8/25 (32)	13/25 (52)	9/25 (36)

*Values are no. or no. positive/no. tested (%). †Fecal samples excluded. The most recent 25 case-patients per hospital district checked. Medical records not available for 6 case-patients. Kuopio last 3 mo of probiotic use, others last 7 d. ‡Medical records not available for 2 case-patients.

### Controls

The controls for the fungemia case-patients (n = 76) were mostly bacteremic (n = 65), but there were 5 case-patients infected with *Candida* sp. Medical records were not available for 6 control case-patients. Median age for this group was 70 years (vs. 68 years for case-patients), 70% were males (versus 63% for the case-patients), 47% had digestive tract disease (vs. 59% of the case-patients), and 17% had a malignancy (vs. 13% of the case-patients) (data were available for 64 case-patients). Use of *S. cerevisiae* var. *boulardii* probiotic by the *Saccharomyces* fungemia group was 43% compared with 5% (4/76) for the control group (odds ratio [OR] 14, 95% CI 4–44).

Microbes detected for controls who had nonblood cultures (n = 123) were also mostly bacteria (n = 97), but *Candida* sp. or other yeasts (n = 51; with or without a concomitant bacterial finding) were more common than in blood cultures. Median age for this group was 65 years (vs. 64 years for case-patients), 44% were males (vs. 59% for case-patients), 70% had digestive tract disease (vs. 69% of case-patients), and 28% had a malignancy (vs. 38% of case-patients) (data on underlying diseases were available for 100 controls). Use of *S. cerevisiae* var. *boulardii* probiotic was 19% (24/125) in the *Saccharomyces* culture-positive group compared with 2% (3/123) for the control group (OR 10, 95% CI 3–32).

## Discussion

We report 20 cases of *Saccharomyces* fungemia in patients who used *S. cerevisiae* var. *boulardii* probiotic. These cases have increased the number of cases reported in the literature by 34%.

We evaluated 125 of 1,153 patients who had a nonblood *Saccharomyces* culture finding and confirmed use of *S. cerevisiae* var. *boulardii* probiotic by 19% of these case-patients. To our knowledge, the magnitude of findings other than fungemia has not been reported. Although some of these nonblood findings might represent colonization, positive *Saccharomyces* cultures led to a change in antimicrobial drugs for 44 (35%) patients who had evaluated cases.

We also evaluated use of *S. cerevisiae* var. *boulardii* probiotic for patients who had a *Saccharomyces* culture finding and compared it with that of a control group who had different positive blood and nonblood cultures and were in the same ward around the same time. The *Saccharomyces* fungemia patients had an OR of 14 and nonblood culture-positive patients had an OR of 10 for use of this probiotic compared with respective controls. Moreover, case-patient 7 (Table 1) is an example of a patient in whom probiotic use unequivocally caused the fungemia. The patient had had a percutaneous endoscopic gastrostomy feeding tube inserted 2 days before the fungemia, had septic shock, and then died. The probiotic was administered at least once through the tube, and the tip of the tube was unintentionally displaced from its ventricular position, leading to an accidental peritoneal application of the probiotic.

*Saccharomyces* fungemias occurred most often in patients who had diseases of the gastrointestinal tract (59%). This finding is nearly identical to the amount reported in a meta-analysis (58%) (*9*). Furthermore, there are reports of *Saccharomyces* fungemias in patients not given pretreatment with a *S. cerevisiae* var. *boulardii* probiotic that have been believed to have been derived from contaminated central venous catheters (*26**–**28*).

Bacteremias and fungemias have not been encountered in clinical trials with probiotics in general. There were probiotic studies conducted with susceptible patients who did not have blood culture findings and who had hepatic encephalopathy (*29*). However, serious concurrent conditions have usually been an exclusion criterion; thus, the safety profile remains unclear. Furthermore, there are other reported safety issues with probiotics, such as contamination of a probiotic supplement with a pathogenic microbe or possible transfer of an antimicrobial drug resistance gene from a probiotic microbe to pathogenic microbes (*30**–**32*).

Regarding the benefits of probiotics, is there evidence showing that adults should use *S. cerevisiae* var. *boulardii* probiotic in conjunction with antimicrobial drugs to prevent *Clostridioides difficile* infections (CDIs) that cause diarrhea? A meta-analysis during 2017 combined *S. cerevisiae* var. *boulardii* probiotic studies conducted in adult populations to prevent CDIs (*33*). There were 5 studies. All studies had a low number of CDIs (15 cases of CDI in control groups) and all had nonsigficant results (pooled risk ratio 0.63, 95% CI 0.29–1.37).

Currently, several companies sell *S. cerevisiae* var. *boulardii* yeast, but total consumption of this yeast in Finland is not known. Thus, we are not able to relate our study results to its use. However, nationwide consumption of probiotics does not reflect the risk for fungemia or nonblood culture findings, or use of this probiotic by susceptible patients in hospitals. Cautious use of *S. cerevisiae* var. *boulardii* probiotic in gastrointestinal surgery wards would probably be one of the most effective ways to decrease these culture findings.

Moreover, the benefits for the indication for which the probiotic is used need to be established. There are 2 recent US guidelines on administration of probiotics in general for primary prevention of CDI. The first guideline states that there are insufficient data to recommend the administration (*34*), and the second guideline states that in certain circumstances certain probiotics could be used, but the quality of evidence is low (*35*).

The first limitation of this study is that we were not able to obtain microbiological evidence that the fungal infections were caused by the *S. cerevisiae* var. *boulardii* probiotic strain and not by another strain. Furthermore, the retrospective design using medical records can lead to a bias in reporting. Only confirmed use of probiotics was reported in this study, and case-patients whose medical records were not available were defined as not using a probiotic. Thus, the percentage of probiotic users was the minimum estimate in all groups. All medications given to patients in the wards were documented in medical records of patients, but patients might have used these medications before they were admitted to the hospital. Moreover, most patients who had fungemia were given bacterial antimicrobial drugs, which could have decreased the routine of taking blood cultures. Recall bias (e.g., the *S. cerevisiae* var. *boulardii* probiotic would have been mentioned in the medical charts more often in case-patients than in control-patients because of *Saccharomyces* culture finding) was not a limitation in this study. For all but 1 case-patient, the probiotic was recorded in the charts before the culture result was complete.

*S. cerevisiae* var. *boulardii* probiotics are not recommended for patients who have indwelling catheters, are immunocompromised, or are critically ill. Our results indicate that use of *S. cerevisiae* var. *boulardii* probiotics should also be considered carefully for patients whose gastrointestinal tract integrity might be compromised. Furthermore, more data are needed to elucidate the health benefits of *S. cerevisiae* var. *boulardii* probiotics in general .
